# Supplementary material for: Modeling glioblastoma heterogeneity as a dynamic network of cell states
Source: Mol Syst Biol. 2021 Sep 16;17(9):e10105. doi: 10.15252/msb.202010105 (PMC8444284; doi:10.15252/msb.202010105)
Supplement: Supplementary file 6 — Source Data for Figure 5 [file MSB-17-e10105-s004.zip › Figure5A_sourcedata/GSEA_3071/nosigdb_stateIV.GseaPreranked.1622795164626/DOROSZKO_U3054MG_CLUSTER_2.html]

Details for gene set DOROSZKO\_U3054MG\_CLUSTER\_2[GSEA]

|  || Dataset | state53071 |
| Phenotype | NoPhenotypeAvailable |
| Upregulated in class | na\_neg |
| GeneSet | DOROSZKO\_U3054MG\_CLUSTER\_2 |
| Enrichment Score (ES) | -0.48398256 |
| Normalized Enrichment Score (NES) | -2.9932845 |
| Nominal p-value | 0.0 |
| FDR q-value | 0.0 |
| FWER p-Value | 0.0 |
Table: GSEA Results Summary

  

Fig 1: Enrichment plot: DOROSZKO\_U3054MG\_CLUSTER\_2      
 Profile of the Running ES Score & Positions of GeneSet Members on the Rank Ordered List

  

| PROBE | GENE SYMBOL | GENE\_TITLE | RANK IN GENE LIST | RANK METRIC SCORE | RUNNING ES | CORE ENRICHMENT || 1 | PDGFRA |  |  | 1 | 2.924 | 0.0104 | No |
| 2 | DCX |  |  | 4 | 2.752 | 0.0199 | No |
| 3 | NRP2 |  |  | 9 | 2.539 | 0.0283 | No |
| 4 | CLU |  |  | 27 | 2.064 | 0.0324 | No |
| 5 | SCRG1 |  |  | 28 | 2.056 | 0.0398 | No |
| 6 | CXCL14 |  |  | 34 | 2.003 | 0.0461 | No |
| 7 | NGFR |  |  | 41 | 1.933 | 0.0518 | No |
| 8 | ADAMTS9 |  |  | 57 | 1.807 | 0.0554 | No |
| 9 | IGFBP3 |  |  | 59 | 1.794 | 0.0617 | No |
| 10 | TCF4 |  |  | 96 | 1.551 | 0.0601 | No |
| 11 | SPRY1 |  |  | 103 | 1.523 | 0.0644 | No |
| 12 | FRMD4B |  |  | 106 | 1.519 | 0.0695 | No |
| 13 | COL1A2 |  |  | 137 | 1.425 | 0.0687 | No |
| 14 | RASSF2 |  |  | 140 | 1.400 | 0.0733 | No |
| 15 | SULF2 |  |  | 146 | 1.384 | 0.0773 | No |
| 16 | ZFHX4 |  |  | 166 | 1.335 | 0.0784 | No |
| 17 | CDK6 |  |  | 178 | 1.299 | 0.0809 | No |
| 18 | GAD1 |  |  | 190 | 1.281 | 0.0833 | No |
| 19 | PLPP3 |  |  | 198 | 1.264 | 0.0865 | No |
| 20 | SOX9 |  |  | 214 | 1.223 | 0.0879 | No |
| 21 | SLC5A3 |  |  | 229 | 1.188 | 0.0894 | No |
| 22 | ITPR1 |  |  | 235 | 1.179 | 0.0927 | No |
| 23 | RGS16 |  |  | 237 | 1.178 | 0.0968 | No |
| 24 | C16orf45 |  |  | 238 | 1.177 | 0.1010 | No |
| 25 | SPRY4 |  |  | 304 | 1.030 | 0.0918 | No |
| 26 | PLXDC1 |  |  | 323 | 1.000 | 0.0918 | No |
| 27 | AC009041.2 |  |  | 335 | 0.985 | 0.0932 | No |
| 28 | CTTNBP2 |  |  | 369 | 0.942 | 0.0900 | No |
| 29 | CAPN5 |  |  | 383 | 0.923 | 0.0908 | No |
| 30 | ZEB1 |  |  | 385 | 0.920 | 0.0939 | No |
| 31 | TGIF1 |  |  | 402 | 0.906 | 0.0940 | No |
| 32 | KLHL13 |  |  | 412 | 0.893 | 0.0954 | No |
| 33 | C11orf96 |  |  | 423 | 0.880 | 0.0966 | No |
| 34 | PLEKHH2 |  |  | 443 | 0.860 | 0.0959 | No |
| 35 | DAAM1 |  |  | 444 | 0.860 | 0.0990 | No |
| 36 | TRIL |  |  | 445 | 0.859 | 0.1021 | No |
| 37 | OLFM2 |  |  | 461 | 0.847 | 0.1022 | No |
| 38 | ANTXR1 |  |  | 462 | 0.847 | 0.1053 | No |
| 39 | FAM198B |  |  | 486 | 0.825 | 0.1037 | No |
| 40 | MRAS |  |  | 491 | 0.823 | 0.1058 | No |
| 41 | TLE1 |  |  | 494 | 0.822 | 0.1084 | No |
| 42 | SHROOM3 |  |  | 509 | 0.811 | 0.1086 | No |
| 43 | TMEM59L |  |  | 539 | 0.778 | 0.1056 | No |
| 44 | HGF |  |  | 558 | 0.758 | 0.1047 | No |
| 45 | CDKN1C |  |  | 565 | 0.751 | 0.1063 | No |
| 46 | ST8SIA4 |  |  | 567 | 0.751 | 0.1088 | No |
| 47 | FGFR1 |  |  | 602 | 0.726 | 0.1046 | No |
| 48 | STIM2 |  |  | 639 | 0.704 | 0.1000 | No |
| 49 | ANGPTL1 |  |  | 647 | 0.692 | 0.1011 | No |
| 50 | TLE2 |  |  | 685 | 0.667 | 0.0961 | No |
| 51 | SEZ6L2 |  |  | 745 | 0.635 | 0.0867 | No |
| 52 | GPR162 |  |  | 784 | 0.611 | 0.0813 | No |
| 53 | JAM2 |  |  | 816 | 0.596 | 0.0773 | No |
| 54 | NES |  |  | 923 | 0.552 | 0.0581 | No |
| 55 | FARP1 |  |  | 931 | 0.549 | 0.0587 | No |
| 56 | PLPPR5 |  |  | 941 | 0.546 | 0.0589 | No |
| 57 | SSBP4 |  |  | 965 | 0.537 | 0.0563 | No |
| 58 | MRC2 |  |  | 969 | 0.535 | 0.0576 | No |
| 59 | VEGFA |  |  | 1010 | 0.518 | 0.0515 | No |
| 60 | C1R |  |  | 1072 | 0.502 | 0.0412 | No |
| 61 | FAM89B |  |  | 1080 | 0.500 | 0.0416 | No |
| 62 | MAPK8IP1 |  |  | 1124 | 0.486 | 0.0348 | No |
| 63 | KCNE4 |  |  | 1194 | 0.468 | 0.0227 | No |
| 64 | NBEA |  |  | 1239 | 0.454 | 0.0156 | No |
| 65 | ADAM22 |  |  | 1307 | 0.439 | 0.0038 | No |
| 66 | EFEMP2 |  |  | 1311 | 0.438 | 0.0048 | No |
| 67 | ATP6V0A1 |  |  | 1320 | 0.436 | 0.0048 | No |
| 68 | STARD13 |  |  | 1350 | 0.428 | 0.0005 | No |
| 69 | CTDSPL |  |  | 1359 | 0.426 | 0.0005 | No |
| 70 | BMP7 |  |  | 1368 | 0.423 | 0.0004 | No |
| 71 | CLEC11A |  |  | 1378 | 0.421 | 0.0001 | No |
| 72 | GALNT2 |  |  | 1439 | 0.406 | -0.0103 | No |
| 73 | IL17RD |  |  | 1446 | 0.403 | -0.0101 | No |
| 74 | CPVL |  |  | 1529 | 0.382 | -0.0250 | No |
| 75 | RAMP2 |  |  | 1537 | 0.381 | -0.0251 | No |
| 76 | TMEM158 |  |  | 1588 | 0.369 | -0.0337 | No |
| 77 | TCEAL5 |  |  | 1630 | 0.362 | -0.0406 | No |
| 78 | LRRC17 |  |  | 1653 | 0.358 | -0.0437 | No |
| 79 | RGL1 |  |  | 1717 | 0.347 | -0.0550 | No |
| 80 | CXCL12 |  |  | 1788 | 0.333 | -0.0677 | No |
| 81 | DCLK1 |  |  | 1860 | 0.321 | -0.0807 | No |
| 82 | ETV4 |  |  | 1937 | 0.307 | -0.0947 | No |
| 83 | CARMIL1 |  |  | 1958 | 0.304 | -0.0976 | No |
| 84 | GLCE |  |  | 2028 | 0.292 | -0.1103 | No |
| 85 | NAB1 |  |  | 2031 | 0.292 | -0.1097 | No |
| 86 | GLT8D1 |  |  | 2071 | 0.287 | -0.1164 | No |
| 87 | AGAP2 |  |  | 2072 | 0.287 | -0.1154 | No |
| 88 | ZDBF2 |  |  | 2104 | 0.283 | -0.1205 | No |
| 89 | SHOX2 |  |  | 2186 | 0.269 | -0.1357 | No |
| 90 | CSRNP3 |  |  | 2188 | 0.269 | -0.1349 | No |
| 91 | CHN1 |  |  | 2221 | 0.266 | -0.1403 | No |
| 92 | ABI3BP |  |  | 2223 | 0.265 | -0.1396 | No |
| 93 | TNFRSF1A |  |  | 2234 | 0.264 | -0.1406 | No |
| 94 | CHSY1 |  |  | 2288 | 0.257 | -0.1502 | No |
| 95 | TSHZ2 |  |  | 2517 | -0.272 | -0.1947 | No |
| 96 | EMILIN1 |  |  | 2542 | -0.274 | -0.1985 | No |
| 97 | NUCB2 |  |  | 2583 | -0.280 | -0.2055 | No |
| 98 | CDC42EP5 |  |  | 2589 | -0.281 | -0.2054 | No |
| 99 | WFDC1 |  |  | 2618 | -0.285 | -0.2100 | No |
| 100 | TNFAIP2 |  |  | 2625 | -0.285 | -0.2102 | No |
| 101 | S1PR2 |  |  | 2631 | -0.286 | -0.2101 | No |
| 102 | GUCY1B1 |  |  | 2642 | -0.288 | -0.2111 | No |
| 103 | DOCK9 |  |  | 2650 | -0.290 | -0.2114 | No |
| 104 | PTCH2 |  |  | 2661 | -0.291 | -0.2124 | No |
| 105 | CRABP2 |  |  | 2691 | -0.294 | -0.2171 | No |
| 106 | STX8 |  |  | 2711 | -0.295 | -0.2198 | No |
| 107 | ECM2 |  |  | 2780 | -0.303 | -0.2323 | No |
| 108 | ASPH |  |  | 2781 | -0.303 | -0.2312 | No |
| 109 | LRRN4CL |  |  | 2805 | -0.306 | -0.2346 | No |
| 110 | TNFRSF12A |  |  | 2890 | -0.318 | -0.2502 | No |
| 111 | PODNL1 |  |  | 3043 | -0.338 | -0.2793 | No |
| 112 | FZD2 |  |  | 3199 | -0.360 | -0.3089 | No |
| 113 | LAMA2 |  |  | 3235 | -0.366 | -0.3146 | No |
| 114 | COL18A1 |  |  | 3266 | -0.370 | -0.3192 | No |
| 115 | FHL3 |  |  | 3274 | -0.371 | -0.3193 | No |
| 116 | CDC42EP2 |  |  | 3294 | -0.374 | -0.3217 | No |
| 117 | OLFML2B |  |  | 3411 | -0.392 | -0.3434 | No |
| 118 | CDR2 |  |  | 3486 | -0.407 | -0.3567 | No |
| 119 | TSPAN5 |  |  | 3515 | -0.412 | -0.3608 | No |
| 120 | TRIM22 |  |  | 3563 | -0.421 | -0.3686 | No |
| 121 | CYGB |  |  | 3579 | -0.423 | -0.3701 | No |
| 122 | CDC42EP3 |  |  | 3608 | -0.430 | -0.3741 | No |
| 123 | GEM |  |  | 3609 | -0.430 | -0.3725 | No |
| 124 | TENT5A |  |  | 3650 | -0.435 | -0.3789 | No |
| 125 | PDE5A |  |  | 3672 | -0.438 | -0.3815 | No |
| 126 | PLIN3 |  |  | 3703 | -0.446 | -0.3859 | No |
| 127 | HES4 |  |  | 3779 | -0.461 | -0.3992 | No |
| 128 | ITGA5 |  |  | 3864 | -0.480 | -0.4142 | No |
| 129 | GLI2 |  |  | 3866 | -0.480 | -0.4127 | No |
| 130 | SAV1 |  |  | 3875 | -0.481 | -0.4125 | No |
| 131 | COL21A1 |  |  | 3888 | -0.484 | -0.4132 | No |
| 132 | MIR222HG |  |  | 3889 | -0.485 | -0.4114 | No |
| 133 | SCPEP1 |  |  | 3903 | -0.487 | -0.4122 | No |
| 134 | ID1 |  |  | 3973 | -0.504 | -0.4242 | No |
| 135 | AFDN |  |  | 3998 | -0.510 | -0.4271 | No |
| 136 | HIC1 |  |  | 4002 | -0.511 | -0.4259 | No |
| 137 | ECE1 |  |  | 4028 | -0.520 | -0.4290 | No |
| 138 | COL8A1 |  |  | 4061 | -0.527 | -0.4334 | No |
| 139 | ODF2L |  |  | 4065 | -0.528 | -0.4321 | No |
| 140 | HTRA3 |  |  | 4114 | -0.544 | -0.4397 | No |
| 141 | PLAC9 |  |  | 4137 | -0.551 | -0.4421 | No |
| 142 | ISLR |  |  | 4155 | -0.556 | -0.4435 | No |
| 143 | NUPR1 |  |  | 4156 | -0.556 | -0.4415 | No |
| 144 | KLF5 |  |  | 4165 | -0.559 | -0.4411 | No |
| 145 | TMEM263 |  |  | 4177 | -0.561 | -0.4412 | No |
| 146 | ASPN |  |  | 4226 | -0.574 | -0.4487 | No |
| 147 | EDNRA |  |  | 4232 | -0.575 | -0.4476 | No |
| 148 | KCTD9 |  |  | 4234 | -0.575 | -0.4458 | No |
| 149 | ABCA5 |  |  | 4264 | -0.586 | -0.4494 | No |
| 150 | PYCR1 |  |  | 4321 | -0.603 | -0.4584 | No |
| 151 | IGFBP7 |  |  | 4331 | -0.607 | -0.4580 | No |
| 152 | RFLNB |  |  | 4345 | -0.613 | -0.4584 | No |
| 153 | SNED1 |  |  | 4348 | -0.614 | -0.4566 | No |
| 154 | PKDCC |  |  | 4376 | -0.627 | -0.4597 | No |
| 155 | ATP2B1 |  |  | 4385 | -0.631 | -0.4590 | No |
| 156 | YIPF5 |  |  | 4389 | -0.632 | -0.4573 | No |
| 157 | YBX3 |  |  | 4405 | -0.637 | -0.4580 | No |
| 158 | OMD |  |  | 4432 | -0.646 | -0.4608 | No |
| 159 | BNC2 |  |  | 4450 | -0.654 | -0.4619 | No |
| 160 | VCAN |  |  | 4464 | -0.661 | -0.4621 | No |
| 161 | TLN2 |  |  | 4475 | -0.667 | -0.4616 | No |
| 162 | ARHGAP10 |  |  | 4486 | -0.672 | -0.4612 | No |
| 163 | DNM1 |  |  | 4528 | -0.696 | -0.4669 | No |
| 164 | PPIC |  |  | 4546 | -0.703 | -0.4677 | No |
| 165 | SYT1 |  |  | 4552 | -0.707 | -0.4662 | No |
| 166 | TBC1D2B |  |  | 4565 | -0.714 | -0.4660 | No |
| 167 | SLC2A3 |  |  | 4629 | -0.754 | -0.4758 | No |
| 168 | PCDH18 |  |  | 4660 | -0.769 | -0.4790 | No |
| 169 | ID3 |  |  | 4686 | -0.788 | -0.4811 | Yes |
| 170 | P3H1 |  |  | 4688 | -0.788 | -0.4785 | Yes |
| 171 | COL5A3 |  |  | 4689 | -0.789 | -0.4756 | Yes |
| 172 | IGFBP6 |  |  | 4693 | -0.790 | -0.4734 | Yes |
| 173 | NR3C1 |  |  | 4699 | -0.796 | -0.4715 | Yes |
| 174 | PLAGL1 |  |  | 4700 | -0.797 | -0.4686 | Yes |
| 175 | MIR22HG |  |  | 4723 | -0.809 | -0.4701 | Yes |
| 176 | SEC24D |  |  | 4730 | -0.815 | -0.4683 | Yes |
| 177 | PDLIM7 |  |  | 4768 | -0.838 | -0.4727 | Yes |
| 178 | CNN2 |  |  | 4774 | -0.842 | -0.4706 | Yes |
| 179 | ADAMTS12 |  |  | 4780 | -0.843 | -0.4686 | Yes |
| 180 | SERTAD4 |  |  | 4787 | -0.849 | -0.4667 | Yes |
| 181 | S1PR3 |  |  | 4799 | -0.865 | -0.4658 | Yes |
| 182 | LOXL2 |  |  | 4816 | -0.881 | -0.4658 | Yes |
| 183 | SPATS2L |  |  | 4833 | -0.901 | -0.4657 | Yes |
| 184 | LIF |  |  | 4841 | -0.907 | -0.4638 | Yes |
| 185 | PALLD |  |  | 4851 | -0.914 | -0.4623 | Yes |
| 186 | CTGF |  |  | 4852 | -0.915 | -0.4590 | Yes |
| 187 | EBF1 |  |  | 4856 | -0.919 | -0.4563 | Yes |
| 188 | NR2F2 |  |  | 4870 | -0.937 | -0.4555 | Yes |
| 189 | PMAIP1 |  |  | 4875 | -0.940 | -0.4529 | Yes |
| 190 | TENM2 |  |  | 4876 | -0.940 | -0.4495 | Yes |
| 191 | TMEM98 |  |  | 4880 | -0.945 | -0.4467 | Yes |
| 192 | ACTN1 |  |  | 4896 | -0.961 | -0.4462 | Yes |
| 193 | KDELR3 |  |  | 4912 | -0.981 | -0.4456 | Yes |
| 194 | MDFIC |  |  | 4941 | -1.004 | -0.4476 | Yes |
| 195 | EPB41L2 |  |  | 4974 | -1.051 | -0.4501 | Yes |
| 196 | TMEM100 |  |  | 4976 | -1.053 | -0.4465 | Yes |
| 197 | EMP1 |  |  | 4977 | -1.056 | -0.4427 | Yes |
| 198 | ATP13A2 |  |  | 4988 | -1.072 | -0.4408 | Yes |
| 199 | NTN4 |  |  | 4991 | -1.079 | -0.4373 | Yes |
| 200 | PRKG1 |  |  | 4998 | -1.089 | -0.4346 | Yes |
| 201 | PIM1 |  |  | 5002 | -1.095 | -0.4312 | Yes |
| 202 | NOTCH3 |  |  | 5003 | -1.098 | -0.4273 | Yes |
| 203 | CLMP |  |  | 5009 | -1.111 | -0.4242 | Yes |
| 204 | FAM114A1 |  |  | 5011 | -1.112 | -0.4204 | Yes |
| 205 | EVA1A |  |  | 5030 | -1.155 | -0.4198 | Yes |
| 206 | GBP3 |  |  | 5033 | -1.163 | -0.4160 | Yes |
| 207 | CYTOR |  |  | 5037 | -1.167 | -0.4124 | Yes |
| 208 | FLRT2 |  |  | 5045 | -1.178 | -0.4096 | Yes |
| 209 | GLT8D2 |  |  | 5046 | -1.179 | -0.4053 | Yes |
| 210 | CAVIN1 |  |  | 5050 | -1.185 | -0.4016 | Yes |
| 211 | DLC1 |  |  | 5057 | -1.204 | -0.3984 | Yes |
| 212 | TWIST1 |  |  | 5062 | -1.216 | -0.3949 | Yes |
| 213 | RASL12 |  |  | 5063 | -1.216 | -0.3905 | Yes |
| 214 | PLXDC2 |  |  | 5064 | -1.217 | -0.3861 | Yes |
| 215 | COPZ2 |  |  | 5071 | -1.227 | -0.3828 | Yes |
| 216 | PROCR |  |  | 5072 | -1.228 | -0.3784 | Yes |
| 217 | SRPX2 |  |  | 5085 | -1.252 | -0.3762 | Yes |
| 218 | MFAP2 |  |  | 5087 | -1.259 | -0.3719 | Yes |
| 219 | DUSP1 |  |  | 5090 | -1.265 | -0.3677 | Yes |
| 220 | LSP1 |  |  | 5095 | -1.278 | -0.3639 | Yes |
| 221 | COL5A2 |  |  | 5097 | -1.282 | -0.3595 | Yes |
| 222 | PAWR |  |  | 5102 | -1.300 | -0.3556 | Yes |
| 223 | GLI1 |  |  | 5114 | -1.321 | -0.3530 | Yes |
| 224 | CPED1 |  |  | 5119 | -1.330 | -0.3490 | Yes |
| 225 | PERP |  |  | 5134 | -1.379 | -0.3468 | Yes |
| 226 | SOCS1 |  |  | 5138 | -1.391 | -0.3423 | Yes |
| 227 | ARL4C |  |  | 5141 | -1.392 | -0.3377 | Yes |
| 228 | SERPINF1 |  |  | 5148 | -1.414 | -0.3338 | Yes |
| 229 | LOX |  |  | 5149 | -1.418 | -0.3287 | Yes |
| 230 | RARRES3 |  |  | 5151 | -1.421 | -0.3237 | Yes |
| 231 | LRP1 |  |  | 5152 | -1.437 | -0.3185 | Yes |
| 232 | BGN |  |  | 5160 | -1.486 | -0.3146 | Yes |
| 233 | COL11A1 |  |  | 5169 | -1.526 | -0.3106 | Yes |
| 234 | CREB3L1 |  |  | 5171 | -1.530 | -0.3053 | Yes |
| 235 | NRP1 |  |  | 5177 | -1.553 | -0.3007 | Yes |
| 236 | GPX8 |  |  | 5184 | -1.588 | -0.2961 | Yes |
| 237 | MXRA8 |  |  | 5191 | -1.622 | -0.2915 | Yes |
| 238 | CD248 |  |  | 5192 | -1.622 | -0.2856 | Yes |
| 239 | MYOF |  |  | 5193 | -1.623 | -0.2797 | Yes |
| 240 | COL12A1 |  |  | 5194 | -1.634 | -0.2738 | Yes |
| 241 | BICC1 |  |  | 5205 | -1.677 | -0.2698 | Yes |
| 242 | SCN9A |  |  | 5207 | -1.686 | -0.2639 | Yes |
| 243 | IGFBP4 |  |  | 5211 | -1.701 | -0.2583 | Yes |
| 244 | TFPI |  |  | 5212 | -1.705 | -0.2522 | Yes |
| 245 | OLFML3 |  |  | 5214 | -1.736 | -0.2461 | Yes |
| 246 | CDH11 |  |  | 5218 | -1.759 | -0.2403 | Yes |
| 247 | THBS1 |  |  | 5222 | -1.800 | -0.2344 | Yes |
| 248 | EGFR |  |  | 5233 | -1.904 | -0.2295 | Yes |
| 249 | COL4A2 |  |  | 5235 | -1.917 | -0.2228 | Yes |
| 250 | DCN |  |  | 5237 | -1.928 | -0.2160 | Yes |
| 251 | SNAI2 |  |  | 5238 | -1.940 | -0.2090 | Yes |
| 252 | MSRB3 |  |  | 5241 | -1.963 | -0.2023 | Yes |
| 253 | AHNAK |  |  | 5244 | -1.992 | -0.1955 | Yes |
| 254 | PCOLCE |  |  | 5247 | -2.050 | -0.1885 | Yes |
| 255 | DAB2 |  |  | 5250 | -2.114 | -0.1812 | Yes |
| 256 | BHLHE41 |  |  | 5251 | -2.129 | -0.1735 | Yes |
| 257 | TRAC |  |  | 5252 | -2.140 | -0.1658 | Yes |
| 258 | PDGFRB |  |  | 5253 | -2.141 | -0.1581 | Yes |
| 259 | COLEC12 |  |  | 5255 | -2.152 | -0.1505 | Yes |
| 260 | CFH |  |  | 5256 | -2.163 | -0.1427 | Yes |
| 261 | CARMN |  |  | 5259 | -2.269 | -0.1349 | Yes |
| 262 | PTCH1 |  |  | 5261 | -2.318 | -0.1267 | Yes |
| 263 | FBN1 |  |  | 5263 | -2.334 | -0.1185 | Yes |
| 264 | COL4A1 |  |  | 5270 | -2.478 | -0.1107 | Yes |
| 265 | LUM |  |  | 5271 | -2.487 | -0.1017 | Yes |
| 266 | SSPN |  |  | 5273 | -2.514 | -0.0928 | Yes |
| 267 | COL5A1 |  |  | 5276 | -2.605 | -0.0838 | Yes |
| 268 | ARHGAP29 |  |  | 5279 | -2.693 | -0.0745 | Yes |
| 269 | INHBA |  |  | 5280 | -2.740 | -0.0645 | Yes |
| 270 | FN1 |  |  | 5284 | -3.017 | -0.0542 | Yes |
| 271 | FOXS1 |  |  | 5286 | -3.055 | -0.0434 | Yes |
| 272 | COL3A1 |  |  | 5287 | -3.508 | -0.0307 | Yes |
| 273 | COL6A3 |  |  | 5288 | -3.654 | -0.0175 | Yes |
| 274 | COL1A1 |  |  | 5290 | -4.897 | 0.0000 | Yes |
Table: GSEA details [plain text format]

  

Fig 2: DOROSZKO\_U3054MG\_CLUSTER\_2: Random ES distribution      
 Gene set null distribution of ES for **DOROSZKO\_U3054MG\_CLUSTER\_2**
